# Determining Glomerular Filtration Rate in Homozygous Sickle Cell Disease: Utility of Serum Creatinine Based Estimating Equations

**DOI:** 10.1371/journal.pone.0069922

**Published:** 2013-07-19

**Authors:** Monika R. Asnani, O’Neil Lynch, Marvin E. Reid

**Affiliations:** Sickle Cell Unit, Tropical Medicine Research Institute, University of the West Indies, Mona Campus, Kingston, Jamaica; INSERM, France

## Abstract

**Background:**

Various estimating equations have been developed to estimate glomerular filtration rate (GFR) for use in clinical practice. However, the unique renal physiological and pathological processes that occur in sickle cell disease (SCD) may invalidate these estimates in this patient population. This study aims to compare GFR estimated using common existing GFR predictive equations to actual measured GFR in persons with homozygous SCD. If the existing equations perform poorly, we propose to develop a new estimating equation for use in persons with SCD.

**Methods:**

98 patients with the homozygous SS disease (55 females: 43 males; mean age 34±2.3 years) had serum measurements of creatinine, as well as had GFR measured using ^99m^Tc-DTPA nuclear renal scan. GFR was estimated using the Modification of Diet in Renal Disease (MDRD), Cockcroft-Gault (CG), and the serum creatinine based CKD-EPI equations. The Bland-Altman limit of agreement method was used to determine agreement between measured and estimated GFR values. A SCD-specific estimating equation for GFR (JSCCS-GFR equation) was generated by means of multiple regression via backward elimination.

**Results:**

The mean measured GFR±SD was 94.9±27.4 mls/min/1.73 m^2^ BSA, with a range of 6.4–159.0 mls/min/1.73 m^2^. The MDRD and CG equations both overestimated GFR, with the agreement worsening with higher GFR values. The serum creatinine based CKD-EPI equation performed relatively well, but with a systematic bias of about 45 mls/min. The new equation developed resulted in a better fit to our sickle cell disease data than the MDRD equation.

**Conclusion:**

Current estimating equations, other than the CKD-EPI equation, do not perform very accurately in persons with homozygous SS disease. A fairly accurate estimating equation, suitable for persons with GFR >60 mls/min/1.73 m^2^ has been developed from our dataset and validated within a simulated dataset.

## Introduction

Undoubtedly, as the populations of persons with sickle cell diseases (SCD) live longer, sickle nephropathy and its various manifestations will emerge in larger numbers. Sickle glomerulopathy, which can progress to end stage renal disease, will continue to rise in its prevalence and have increasing contributions to the morbidity and mortality profile of these persons [1], [2]. End stage renal failure is found to be present in about 11% of persons with SCD and tends to rise with age [3], [4].

It is important to be able to detect renal dysfunction in its early stage so that possibly, interventions can delay or stop this dysfunction from worsening to renal failure. Recommendations for clinical practice include screening for albuminuria and estimating glomerular filtration rate (GFR) to monitor renal function in persons especially at risk for renal diseases [5], [6], [7]. Albuminuria is now proposed to be tested by spot urine albumin: creatinine ratios (ACR) instead of the much more cumbersome and potentially inaccurate determinations of protein excretion in 24 hour collections of urine. Estimated GFR (eGFR) is proposed to be used to categorize persons into chronic kidney disease (CKD) stages using the modified serum creatinine based Modification of Diet in Renal Disease (MDRD) equation [8], [9]. The utility of the modified MDRD equation, and in fact other currently used serum creatinine based GFR estimating equations, in persons with SCD is not very clear. Due to the unique renal physiologic processes that occur in SCD [10], [11], it is wise to determine this utility before widespread use of the equations in this population.

In this study we propose to compare GFR levels estimated using the modified MDRD (eGFR_MDRD), Cockcroft-Gault (eGFR_CG), and the serum creatinine based Chronic Kidney Disease Epidemiology Collaboration (eGFR_CKDEPI) equation to GFR levels measured using the radiolabeled 99m-Tecnetium diethylenetriamine pentaacetic acid (99m-Tc DTPA) renal scan (mGFR_DTPA) in adults with SCD. We hypothesize that due to the differences in serum creatinine generation and thereafter handling by the sickle kidney, these equations will not show good limits of agreement in persons with SCD, and we therefore propose to generate a new GFR estimating equation specific for SCD.

## Materials and Methods

The study was conducted in persons with the homozygous SS sickle cell disease from the Jamaica Sickle Cell Cohort Study (JSCCS). The cohort has been followed at the Sickle Cell Unit (SCU) at University of the West Indies in Jamaica since birth and participants are now between the ages of 29 and 39 years. All 98 participants were studied when they were having no acute illness, had not had any blood transfusion in the preceding one month, and when in the non-pregnant state. None of our patients were on Hydroxyurea or a chronic blood transfusion programme at the time of the study.

### Ethics Statement

The study was granted ethical approval by the University of the West Indies/University Hospital of the West Indies Ethical Committee. The study was designed and performed in adherence with the Declaration of Helsinki. Written, informed consent was obtained from all participants involved in the study.

### Procedures

The participants attended the SCU by appointment and were seen by the study nurse. Height, weight and blood pressure were measured with a staidiometer, beam balance and Dinamap™ respectively.

Each person had approximately 10 mls of venous blood sample taken for haematology and biochemistry measurements. Haematological measurements included haemoglobin, white blood cell, platelets, and reticulocyte counts and were performed at the SCU laboratory itself.

Serum and urinary creatinine were measured using the VITROS® 350 Analyser at a private laboratory in Kingston, Jamaica. The VITROS CREA slide method was employed which uses a multi-layered, analytical element coated on a polyester support. This employs an enzymatic method of determining creatinine levels in the ranges of 4–1238 µmol/L (0.05–14.0 mg/dl) in serum and 106– 30631 µmol/L (1.2–346.5 mg/dl) in urine samples. The values assigned to the VITROS Chemistry Products Calibrator Kit for Creatinine are traceable to a Gas Chromatography Isotope Dilution Mass Spectrometry (GC/IDMS) method [12] and National Institute of Standards and Technology (NIST) SRM® 914 creatinine standard reference material. Bilirubin levels upto 342 µmol/L (20 mg/dl) were shown to have nil interference, i.e. <8.8 µmol/L (<0.1 mg/dl) bias; this is especially important in persons with SCD who tend to be hyperbilirubinaemic due to their hyper-haemolytic state.

The participants were all transported to a private nuclear medicine facility for the 99m-Tc DTPA renal scan. Prior to the examination, each patient was well hydrated and renography was carried out with the patient in the supine position with imaging done posteriorly. 5mCi of Technetium 99 m labelled with DTPA was injected as a bolus. The protocol used for acquisition and analysis is Gates GFR (DTPA) Adult Renal Protocol. The Acquisition parameters include the input of Patient height and weight (body surface area), patient age and sex. Syringe counts were done pre and post radiopharmaceutical injection, and dynamic acquisition was done for a period of 6 mins, using 15 sec per frame for a total of 24 frames. The renogram graphs were generated using an ROI drawn for each kidney, and total, differential and normalised GFR was computer generated.

### Statistical Analysis

All analyses were performed using Stata Software version 11.0 for Windows™ (StataCorp, College Station, TX).

Gender-specific descriptive analyses were performed on the participants. Results were expressed as means with standard deviations (sd).

### Estimating GFR

GFR was estimated using the Cockcroft-Gault (eGFR_CG), the modified Modification of Diet in Renal Disease (eGFR_MDRD), and the serum creatinine based Chronic Kidney Disease Epidemiology Collaboration (eGFR_CKDEPI).

The following equations were utilized:

- **eGFR_CG** =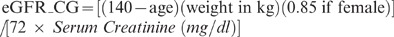

- **eGFR_MDRD** = 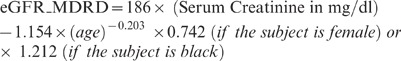

- **eGFR_CKDEPI** ***(Serum Creatinine [Scr] in mg/dl)***

**Female**

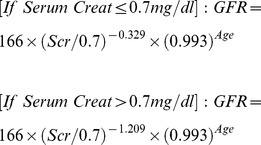



**Male**

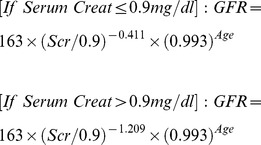



The measured GFR and the eGFR_CG were both adjusted for body surface area (BSA) of 1.73 m^2^; the latter calculated using the DuBois formula.

Pairwise correlations were done between measured GFR and GFR estimated via all 3 existing equations. Significance levels were set at p value<0.01.

The agreement of measured GFR with eGFR_MDRD, eGFR_CG, and eGFR_CKDEPI equations was assessed using the Bland-Altman limit of agreement method.

### Generating a SCD-specific Estimating Equation (Jamaica Sickle Cell Cohort Study GFR Equation: JSCCS-GFR Equation)

Firstly we carried out logarithmic transformation of the following variables: measured GFR, serum creatinine, and height. The candidate predictor variables were age, gender, creatinine and height and were regressed against log-transformed measured GFR. A parsimonious model was obtained by the technique of backward elimination and the p-value for elimination was set at 0.05. Further regression diagnostics were performed. These included the leverage-versus-squared-residual plot and Cook’s distance statistics so as to eliminate potential outliers. These diagnostics tools eliminated 10 observations from our sample which resulted in a satisfactory mean variance inflation factor of 1.29. Additionally, the omitted-variables test and the heteroskedasticity test were done resulting in p-values of 0.8154 and 0.7836 respectively. This yielded the JSCCS-GFR equation with creatinine and height as the significant predictors of measured GFR.

As a validation strategy a simulated data set was generated using Monte Carlo method having the same variance-covariance structure as the original sickle cell disease data. The simulated data consists of 100 observations with equal proportion of males and females. The SCD-specific equation was tested against this simulated data set.

Once again, pairwise correlations were done between GFR estimated from this new equation and all other GFR values. Accuracy of all estimating (the 3 existing as well as the newly generated) equations was assessed by determining the percentage of estimated GFR lying within 30% (P30) and 10% (P10) of the measured GFR from each of the four equations.

## Results

A total of 98 persons (55 Females: 43 Males) with homozygous SS disease from the JSCCS met the inclusion criteria and completed the study. The mean age was 34.0±2.3 years (range: 29.8–38.6 years). The mean measured GFR was 94.91±27.37 mls/min/1.73 m^2^ (range of 6.41–159.01 mls/min/1.73 m^2^), and was normally distributed over this range. There were minimal gender differences where diastolic blood pressure was higher (65.7±9.5 mmHg vs. 60.3±9.3 mmHg, p-value: 0.006) and height was lower (166.7±6.7 cm vs. 172.6±8.0 cm; p-value <0.001) in females.

Table 1 shows the general characteristics of the study sample by gender, the GFR values estimated from various existing equations as well as the DTPA- measured GFR in all participants. All existing estimating equations appear to provide an overestimate of measured GFR. All pairwise correlations were significant at p value<0.01, but of moderate magnitude (Table 2). The measured GFR showed the strongest correlations (r = 0.70) with the newly generated estimating equation.

**Table 1:** General characteristics of participants in GFR study (n = 98).

Variable	Males(n = 43)	Females(n = 55)	p-value
**Age, years**	33.8±2.2	34.1±2.5	0.54
**Weight, Kg**	60.2±12.6	58.6±9.4	0.47
**Height, cm**	172.6±8.0	166.7±6.7	**<0.001**
**Systolic Pressure, mmHg**	108.7±11.9	111.6±13.5	0.28
**Diastolic Pressure, mmHg**	60.3±9.3	65.7±9.5	**0.006**
**Haemoglobin, g/dl**	7.7±1.5	7.3±1.5	0.22
**White blood cells, 10^9^/L**	11.3±3.1	12.0±3.9	0.35
**Platelets, 10^6^/L**	371.7±146.5	417.0±124.1	0.10
**Reticulocytes, %**	11.0±4.2	11.1±3.4	0.83
**Serum Creatinine, µmol/L**	65.8±23.8	72.6±112.5	0.70
**Serum Creatinine, mg/dl**	0.75±0.27	0.82±1.27	0.70
**Measured GFR, mls/min/1.73 m^2^**	94.4±24.5	95.3±29.6	0.87
**eGFR_MDRD, mls/min/1.73 m^2^**	172.7±52.6	159.4±55.9	0.23
**eGFR_CG, mls/min/1.73 m^2^**	129.5±34.3	135.4±44.8	0.48
**eGFR_CKDEPI, mls/min/1.73 m^2^**	140.0±23.9	133.1±29.7	0.22

All results are mean ± SD.

**Table 2:** Pairwise correlations between measured and estimated GFR.

	Measured GFR	eGFR_MDRD	eGFR_CG	eGFR_CKDEPI	JSCCS_GFR
**Measured GFR**	1.0				
**eGFR_MDRD**	0.61	1.0			
**eGFR_CG**	0.59	0.93	1.0		
**eGFR_CKDEPI**	0.66	0.88	0.84	1.0	
**JSCCS_GFR**	0.70	0.89	0.85	0.87	1.0

### Limits of Agreements between Measured and Estimated GFR

**Figure 1:**
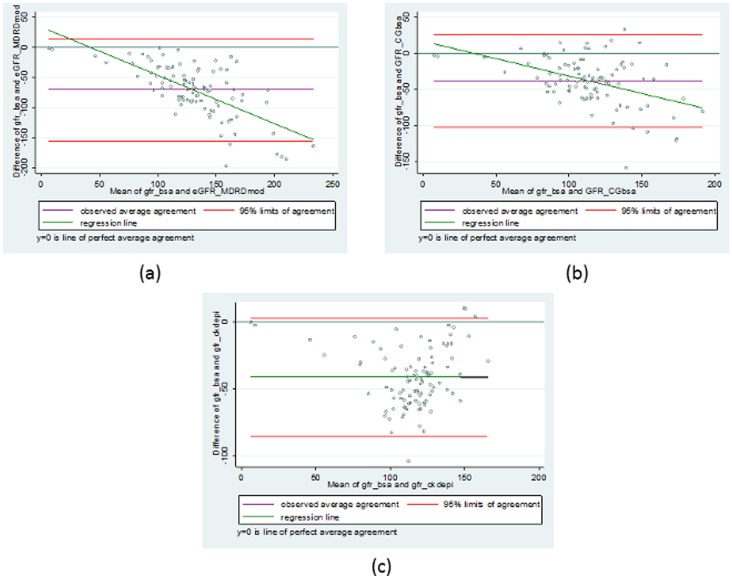
Limits of Agreement Plots: Measured GFR versus (a) MDRD estimate (b) CG estimate and (c) Serum Creatinine based CKD-EPI estimate.

Figure 1 graphically displays the agreement between measured GFR and GFR estimated from the modified MDRD equation (a), CG equation (b), and the serum creatinine based CKD-EPI equation (c). The MDRD overestimated the GFR by a mean of 70.4 mls/min and this difference increased as GFR increases (correlation between difference and mean is 0.69). The lower and upper limits of agreement were −155.8 and 15.1 mls/min. The CG overestimated the GFR by a mean of 37.9 mls/min and this difference increased as GFR increases (correlation between difference and mean is 0.44). The lower and upper limits of agreement were −102.4 and 26.7 mls/min. The normal plot of differences from these two equations was also non-normal (not shown). The serum creatinine based CKD-EPI overestimated the GFR by a mean of 41.2 mls/min; and correlation between difference and mean was 0.002, which implies very good concordance between the two methods. The lower and upper limits of agreement were −85.2 and 2.8 mls/min. The observed average agreement and regression line on the ‘limits of agreement’ plot overlapped perfectly. The normal plot of differences demonstrated near normality, except minor deviations at each extreme of measurements (not shown).

The P30 was low for all three equations: 15.3%, 35.7%, and 27.6% respectively for the MDRD, CG, and CKD-EPI equations; and the P10 was lower: 2.0% for the MDRD, 13.3% for the CG and 10.2% for the CKD-EPI equation (Table 3).

**Table 3:** Bland-Altman method of comparison of measured GFR using ^99m^Tc-DTPA and GFR estimated from existing equations.

Estimating Equation	Mean Difference (Bias)	SD of Diff (Precision)	95% Limits of Agreement		P30* (%) (Accuracy)	P10** (%) (Accuracy)
**MDRD**	−70.4	43.6	−155.8	15.1	15.3	2.0
**CG**	−37.9	32.9	−102.4	26.7	35.7	13.3
**CKD-Epi**	−41.2	22.5	−85.2	2.8	27.6	10.2
**JSCCS_GFR**	−0.67	19.7	−39.2	37.8	82.7	40.8

* P30: Expressed as the percentage of estimated values within 30% of measured values.

** P10: Expressed as the percentage of estimated values within 10% of measured values.

### New GFR Estimating Equation for SCD

The resulting estimating equation for SS disease is given by:
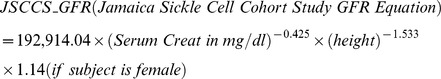



This equation had a better correlation (r = 0.70) with the measured GFR than all other existing equations. Its accuracy also was greater: P30 of 82.7% and P10 of 40.8%.

**Figure 2:**
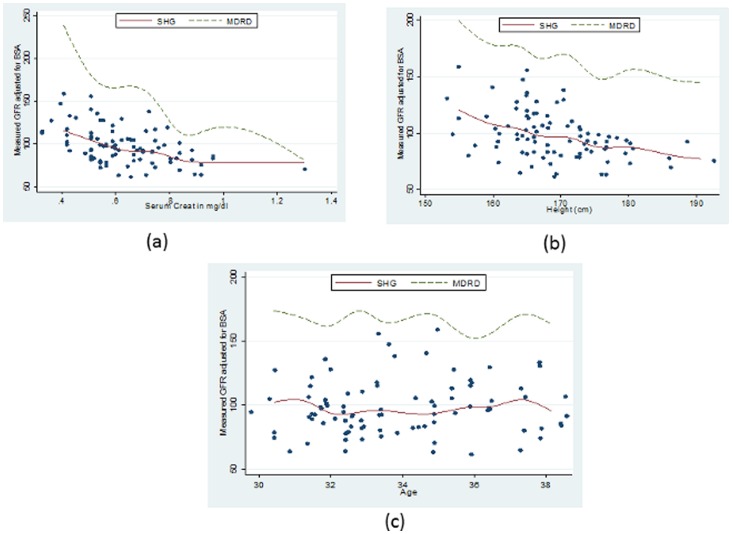
Comparison of the new model JSCCS_GFR (GFR as a function of (a)Serum Creatinine, (b) Height and (c) Gender) from the sickle cell disease data with the MDRD model.

**Figure 3:**
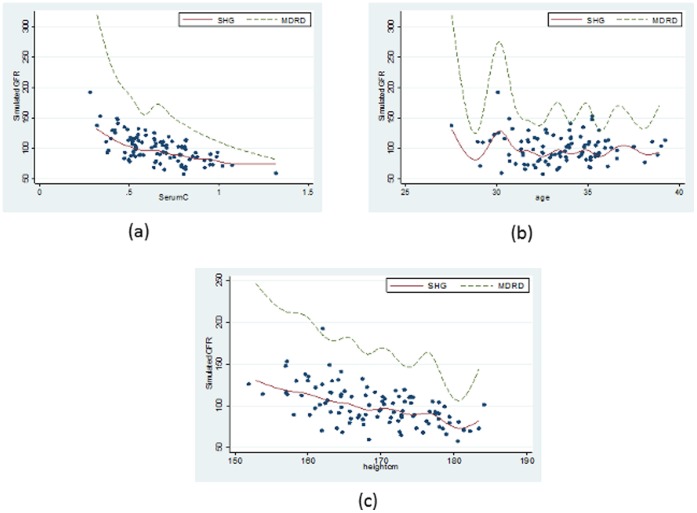
Comparison of the new model JSCCS_GFR (GFR as a function of (a)Serum Creatinine, (b) Height and (c) Gender) from the simulated sickle cell disease data with the MDRD model.

This model resulted in a better fit to our sickle cell disease data than does the MDRD equation which can be seen in Figure 2, which shows plots of Measured GFR against (a) Serum Creatinine, (b) height and (c) age, while Figure 3 displays plots of Simulated GFR against (a) Serum Creatinine, (b) height and (c) age.

Figure 2 depicts how our model **JSCCS_GFR** (GFR as a function of Serum Creatinine, Height and Gender) from the sickle cell data compares with the MDRD model.

Figure 3 depicts how our model **JSCCS_GFR** (GFR as a function of Serum Creatinine, Height and Gender) from the simulated sickle cell data compares with the MDRD model.

## Discussion

The KDOQI guidelines advise clinicians to estimate and monitor GFR using the modified MDRD equation. However as this equation was formulated in persons with CKD (and not in populations with normal renal function), its utility is limited in persons with higher GFR levels. Studies have also shown poor predictive value in various subgroups such as in ill and hospitalized patients [13], in liver cirrhosis [14], in obese patients [15], in diabetics with normal serum creatinine levels [16], etc. Efforts are therefore continuing to develop more accurate equations to have broader utility in diverse populations, and the latest is the development by the CKD-EPI collaboration of the serum creatinine based CKD-EPI equation, a serum Cystatin C based equation, and an equation based on a combination of both these parameters [9], [17], [18].

This study demonstrates that the recommended MDRD and the CG equations tend to overestimate the GFR. The serum creatinine based CKD-EPI, apart from having a positive bias of about 45 mls/min, tends to be the closest in its estimates over a wide range of GFR values.

Despite the understanding among SCD researchers that:

1. there is likely to be tubular dysfunction in SCD both from chronic possible use of non-steroidal anti-inflammatory agents in the management of recurrent pain episodes in persons with SCD, as well as an increased tubular secretion of creatinine [11],
2. there might be differences in creatinine generation due to reduced muscle mass in SCD as well as variability in protein catabolism, hydration status etc. [10],

all of which would lead to distorted levels of serum creatinine and creatinine clearance measurements in urine as well as lead to invalidating current methods to determine renal dysfunction, these methods are being widely used in assessing kidney function in SCD [19], [20], [21], [22], [23], [24], [25]. These studies have included children and adults and have utilized various predictive equations such as MDRD, CG, Schwartz, and CKD-EPI to estimate GFR as a marker of renal function in these studies.

Some effort has been made to determine agreement of certain predictive equations with measured GFR in SCD patients. Most have been in children. The Baby HUG investigators have used measurements using ^99m^Tc-DTPA and found them to show poor correlation with the Schwartz formula [26], but fair correlation with the CKid-Schwartz (Chronic Kidney Disease in Children) formula [27]. Aygun et al [28] also compared in young children GFR measured using ^99m^Tc-DTPA and compared to various creatinine and Cystatin C based estimates and found them all to be fairly lacking.

Haymann et al [22] determined the sensitivity and specificity of the MDRD equations to be good when compared to ^51^Cr-EDTA measurements of GFR in a subset of their adult SS study population. However the method did over-estimate the true GFR by a large amount. Aparicio et al [10] measured GFR using ^51^Cr-EDTA as well and found it to correlate poorly with creatinine clearance methods.

To our knowledge, this is the largest study to date which has aimed to determine clearly the utility of existing recommendations to estimate GFR in persons with homozygous SS disease. Our results have demonstrated that the KDOQI recommendations of using MDRD based GFR estimates may not be applicable in persons with SCD. The most recent CKD-EPI equation shows good promise in this population, and in fact it is becoming clearer even among other populations that these estimates may prove more accurate and in the near future may be adopted for widespread clinical use. Even with the bias that has been demonstrated in this study, the CKD-EPI equation can be recommended to closely monitor changes in GFR.

We have also developed a new equation specific to persons with SCD that performs better than the other equations, at least in those with GFR levels above 60 mls/min/1.73 m^2^. The equation is based on serum creatinine and height of the persons, and is gender specific. One of the main limitations of the study in that it was conducted in a very narrow age group of young adults, ranging from about 29 years to 39 years.

This study, which is limited by its cross-sectional design, has been conducted in a well-defined cohort of patients with SCD that have been very closely followed since birth. As this cohort study is ongoing, longitudinal data collection and application of this equation will allow for its further refinement.

### Conclusions

In conclusion, we state that the use of existing serum creatinine based equations to estimate GFR in clinical practice may not be very accurate in persons with SCD. The serum creatinine based CKD-EPI equation may however prove to be useful and be more readily applied for clinical use.

A closer estimate of GFR, especially in those with GFR levels above 60 mls/min/1.73 m^2^ can be made by using the proposed equation that has been generated in this study. Work will need to be done to study its utility further before wide spread use can be recommended.
