# Correction: Determining Glomerular Filtration Rate in Homozygous Sickle Cell Disease: Utility of Serum Creatinine Based Estimating Equations

**DOI:** 10.1371/journal.pone.0228211

**Published:** 2020-01-16

**Authors:** The PLOS ONE Staff

In the New GFR Estimating Equation for SCD subsection of the Results, there is an error in the equation. The text inside the fourth set of parentheses is incorrect. It should read: (if subject is male). The publisher apologizes for the error. Please view the complete, correct equation here:

$$\begin{array}{l} JSCCS\_GFR\left(Jamaica\;Sickle\;Cell\;Cohort\;Study\;GFR\;Equation\right)=\\ 192,914.04\times {\left(Serum\;Creat\;in\;mg/dl\right)}^{-0.425}\times {\left(height\right)}^{-1.533}\times 1.14\left(if\;subject\;is\;male\right) \end{array}$$
